# Mpox seroprevalence among sex workers and men who have sex with men preceding sustained urban outbreak in the Democratic Republic of the Congo

**DOI:** 10.1017/S0950268826101861

**Published:** 2026-06-23

**Authors:** Patrick K. Mukadi, Sydney Merritt, Antoine Nkuba-Ndaye, Megan Halbrook, Yvon Anta, Tavia Bodisa Matamu, Emmanuel Hasivirwe Vakaniaki, Daniel Mukadi-Bamuleka, Ryan J. Harrigan, Lygie Lunyanga, Cris Kacita, Jean Paul Kompany, Sylvie Linsuke, Candice Lemaille, Marie Clotilde Inaka, Ahidjo Ayouba, Nicole A. Hoff, Jason Kindrachuk, Lisa E. Hensley, Martine Peeters, Placide Mbala-Kingebeni, Steve Ahuka-Mundeke, Anne W. Rimoin

**Affiliations:** 1 Epidemiology and Global Health, Institut National de Recherche Biomédicale, Congo, The Democratic Republic of the; 2 Service de Microbiologie, Département de Biologie Médicale, Cliniques Universitaires de Kinshasa, Universite de Kinshasa Faculte de Medecine, Kinshasa, Congo, The Democratic Republic of the; 3 Epidemiology, University of California Los Angeles Jonathan and Karin Fielding School of Publi, Los Angeles, USA; 4 TransVIHMI, Universite de Montpellier, INSERM, Institut pour le Recherche et Developpement (IRD), Montpellier, France; 5 Virology, Institut National de Recherche Biomedicale, Kinshasa, Congo, The Democratic Republic of the; 6 Rodolphe Merieux Institut National de Recherche Biomedicale (INRB) Laboratory, Goma, Congo, The Democratic Republic of the; 7 Center for Tropical Research, UCLA Institute of the Environment and Sustainability, University of California Los Angeles, Los Angeles, USA; 8 National Program for the Control of Mpox and Viral Hemorrhagic Fevers, Government of the Democratic Republic of the Congo Ministry of Health, Kinshasa, Congo, The Democratic Republic of the; 9 Medical Microbiology & Infectious Diseases, University of Manitoba Max Rady College of Medicine, Winnipeg, Canada; 10 National Program for HIV/AIDS, Ministry of Health, Kinshasa, Kinshasa, Democratic Republic of the Congo; 11 National Microbiology Laboratory Branch, Public Health Agency of Canada, Winnipeg, Canada; 12 Zoonotic and Emerging Disease Research Unit (ZEDRU), United States Department of Agriculture, Agriculture Research Services (USDA-ARS), Manhattan, USA

**Keywords:** DR Congo, high risk populations, Mpox, MPXV Clade I, seroprevalence, urban centres

## Abstract

Mpox has evolved into a global public health concern following rapid geographic expansion and altered clinical and epidemiological characteristics. The 2022 outbreak of monkeypox virus (MPXV) marked the first widespread expansion to non-endemic regions, particularly among men who have sex with men (MSM). In the Democratic Republic of the Congo (DRC), where Clade I MPXV is endemic, data on urban exposures are limited. We evaluated serologic evidence of exposure using a four-plex MPXV assay among 1,823 participants from two major DRC cities, Kinshasa and Goma, between April and June 2024. Of these participants, 523 (28.7%) self-identified as MSM, 498 (27.3%) as professional sex workers (SW), 107 (5.9%) as MSM and SW, and 695 (38.1%) as other at-risk individuals. Overall, 2.9% of participants were seroreactive. By subpopulation, 4.8%, 2.1%, and 1.7% of at-risk, MSM, and SW participants were seroreactive (*p* = 0.0086). Classification tree modelling identified age as the strongest predictor of reactivity, with higher MPXV seroreactivity in older participants. These data provide evidence of mpox seroreactivity prior to sustained human-to-human transmission in urban settings. Understanding exposure dynamics is critical for anticipating re-emergence of mpox in global cities where interpersonal networks may sustain transmission even in the absence of zoonotic spillover.

## Introduction

Mpox (previously monkeypox), is a zoonotic viral disease endemic in tropical forested regions of Central and West Africa, with the Democratic Republic of the Congo (DRC) being the most affected country. Since reporting the first human case in 1970 [1], the DRC has experienced substantial increases in mpox incidence, with over 10,000 laboratory-confirmed cases reported in 2024 alone [2–5]. Historically, human mpox incidence has been driven by zoonotic events with self-limiting transmission clusters in remote regions, where rodents were believed to play a key role as a potential reservoir [6].

Genomically, monkeypox virus (MPXV) has been classified as two separate clades: Clade I, endemic in regions of the Congo River Basin, and Clade II, endemic in Western African regions [7]. While studies have shown virulence differences between the two clades, more recent observations from mpox outbreaks have been less certain. Both virus clades have demonstrated transmission through similar zoonotic events, and increasing persistence in human populations, with seemingly context-specific divergence [8]. Although mpox outbreaks had previously been largely confined to endemic regions of Africa, the 2022 global outbreak marked a major epidemiological shift with rapid international spread of Clade IIb among non-endemic regions, and infections concentrated among men who have sex with men (MSM) [9, 10]. Unlike earlier zoonosis-driven outbreaks, transmission during this outbreak was sustained through human-to-human spread within dense sexual networks and the absence of zoonotic exposures [11]. Furthermore, in 2024, a novel sub-lineage of Clade I was identified – Clade Ib – with sustained human-to-human transmission clusters first detected in Kamituga, South Kivu Province, DRC [4]. Subsequent work delineating Clade I MPXV diversity has shown a predominance of zoonotic transmission in DRC related to Clade Ia, historically comprising Clade I, with specific sexual networks and high population density contributing to the continued propagation of mpox (Clade Ib) in urban centres [12].

Today, outbreaks of mpox in urban settings continue to be sporadically reported both in endemic and non-endemic settings; for instance, as of October 2025, Clade I MPXV was confirmed in Los Angeles County, California, USA, in patients with no reported travel to endemic regions, yet later linked to an importation event with evidence of local transmission [13, 14]. While continued mpox emergence and sustained transmission was historically unprecedented in non-endemic settings, these recurrent events highlight the need to examine transmission dynamics in urban centres in mpox-endemic countries to clarify whether similar network-driven mechanisms occur, or whether distinct social, ecological, or virological factors sustain mpox transmission in these regions.

Recent studies have highlighted the increasing role of sexual transmission in mpox outbreaks in the DRC. A notable cluster of Clade Ia infections in March 2023 associated with sexual or intimate contact first suggested that this new mode of transmission was not unique to the Clade IIb variant [15]. In Kamituga, South Kivu Province, an urban outbreak in late 2023 identified 29% of PCR-confirmed cases as female sex workers (SWs), with 61% of suspected cases as sexually active and reporting multiple partners, all supporting the hypothesized role of sexual-contact driven transmission [4]. The prevalence of APOBEC3-mediated mutations in Clade Ib MPXV further suggested ongoing human-to-human transmission [16]. Concurrently, mounting evidence suggests that human-to-human chains are now sustaining outbreaks even in non-endemic regions. Molecular and serological screening in Central Africa and Europe have uncovered both symptomatic and asymptomatic MPXV infections in sexual health clinics, implying that clinical surveillance may vastly underestimate true transmission – particularly within dense sexual networks [17, 18].

Widespread mpox surveillance in the DRC is limited to those symptomatic cases identified at local health centres and reported passively through structures such as the Integrated Disease and Response System and to the National Program for the Control of Mpox and Viral Hemorrhagic Fevers (French acronym, PNLMPX-VHF) [19]. Yet, these systems are resource-limited and support few research-driven, case-specific outbreak investigations. Furthermore, contact-tracing and confirmatory laboratory testing are even more restricted due to historical underfunding and logistical challenges associated with biological specimen collection and appropriate transport [20]. While additional resources were mobilized to conduct active surveillance based at the National Institute for Biomedical Research (French acronym, INRB) during the declared Public Health Emergency of International Concern, this activity was specifically initiated in response to World Health Organization declarations. Given these constraints, seroprevalence studies are valuable approaches to understand the extent of mpox exposure and population immunity and to identify potential risk factors [21, 22].

Considering the epidemiologic shifts of mpox transmission and those sub-populations considered most at-risk globally, as well as the presumed underreporting of mpox in DRC, this study sought to determine prevalence of anti-MPXV antibodies among suspected high-risk populations in urban centres. Specifically, this study assessed seroprevalence among those populations most affected by the 2022 global outbreak, those vulnerable due to dense sexual networks and limited access to health care due to social stigma: MSM and professional SW. Furthermore, sampling also included those populations at increased risk due to zoonotic exposure through their occupation or other reported lifestyle habits. Outside of DRC, previous serosurveillance studies in Kenya have indicated high mpox seropositivity rates in these groups in the absence of reported infections, indicating significant underreporting of urban mpox cases [23].

## Methods

### Population recruitment and sample collection

This cross-sectional study sought to determine the seroprevalence of mpox among key populations in two geographically distinct urban centres of DRC: Kinshasa (Kinshasa Province) and Goma (North Kivu Province). These locations were selected due to their high population density and mobility, factors that elevate the risk of MPXV transmission. Between April and June 2024, participants were recruited from three suspected at-risk sub-populations: self-identified MSM, self-identified SW, and other at-risk members of the general population based on their location and occupation. At the time of enrolment, mpox-specific vaccines were not available for administration. Furthermore, at the time of enrolment, reported MPXV cases in Kinshasa were restricted to self-limiting clusters, while in Goma, Clade Ib MPXV was first detected among individuals travelling to the city or in nearby internally displaced persons camps in May 2024 [24, 25].

To enrol these often socially subtle, at-risk groups, recruitment strategies relied on a combination of time-location and snowball sampling. Peer educators (PEs), who were members of the MSM and SW communities, played a key role in the recruitment process [26]. In collaboration with the National Program for the Fight against HIV/AIDS and Sexually Transmitted Infections (French acronym, PNLS) and PEs, key locations frequented by these populations, such as health facilities, bars, nightclubs, markets, and city entrances, were identified for sampling.

Eligible participants were recruited if they were ≥18 years old and self-identified as either a MSM or SW. Additionally, at PE-directed sampling sites, non-MSM and non-SW were enrolled as a convenience sample of an otherwise at-risk population (ARP); the ARP also included those participants recruited at farms and abattoirs (suspected occupational zoonotic exposure). All participants consented to both survey participation and blood sampling [26]. This study was approved by the Institutional Review Boards of the University of California, Los Angeles (IRB#23–000676), University of Manitoba (HS25837) and the Kinshasa School of Public Health, University of Kinshasa, DRC (ESP/CE/161/2024).

Data collection consisted of two main components: an interviewer-led semi-structured questionnaire, and serum collection at enrolment. The survey instrument, hosted on a secure survey platform server (*SurveyCTO* (Version 2.0), Dobility, Inc), included questions on demographic characteristics (e.g., age, education, marital status), sexual and clinical history, knowledge of mpox transmission, and zoonotic and sexual risk factors. Additionally, trained phlebotomists collected a venous blood sample that was aliquoted in the field into serum, and plasma and buffy coat samples.

### Serologic testing

Serum samples were evaluated for reactivity using a four-plex immunoassay of three synthetic peptides derived from the right terminal region B of MPXV, B21R.179/180, B21R.185/186, and B22R.64/65 [27], as well as the highly-conserved *Poxviridae* peptide, H3L [28, 29]. In brief, each peptide was first conjugated to bovine serum albumin before covalent attachment to magnetic beads with distinct fluorescent signatures (Luminex Corp, Austin, TX, USA). A master-mix bead solution containing Phosphate Buffered Saline (PBS) with 0.75 mol/NaCl, 5% heat-inactivated foetal bovine serum (Gibco-Invitrogen, France), 1% bovine serum albumin, and 0.2% Tween-20 (Sigma-Aldrich, France) was added to each well of 96-well flat-bottom chimney plates (Greiner Bio-One, Germany). Diluted serum samples (1:200) were subsequently added to individual wells in duplicate; following washing steps, a secondary biotin-labelled anti-anti-human IgG antibody (BD-Pharmingen, France) was added. Finally, streptavidin-R-phycoerythrin (Fisher Scientific/Life Technologies, France) was introduced; with antigen-antibody binding quantified as median fluorescence intensity (MFI) per 100 beads using a Magpix instrument (Bio-Rad, France). All sample testing was completed on-site in Kinshasa, DRC.

As described by Kinganda-Lusamaki et al., the mean MFI plus three standard deviations of a 90-person mpox-negative panel was calculated as the respective seroreactivity threshold for each of the MPXV-specific peptides, B21R.179/180, B21R.185/186, and B22R.64/65 [18]. Given the unknown orthopoxvirus vaccination status of the 90-person panel, the cut-off value for anti-H3L reactivity was calculated using the likelihood ratio from a receiver operating characteristic curve (Supporting Information: Supplementary Figure S1 of the original publication [18]).

Seroreactivity results were binarized, calculated by dividing the MFI value of each sample by the corresponding cut-off for each peptide; an index >1 was considered seroreactive. As positivity to any individual peptide of the four-plex was deemed sufficient for a seroreactive result [27], the maximum index value was considered for each sample.

### Statistical analysis

All data analysis was completed with SAS version 9.4 (SAS Institute, Cary, NC) and R version 4.3.3 (R Foundation for Statistical Computing, Vienna, Austria). Descriptive statistics were generated for participant demographics and seroreactivity rates. Unadjusted measures of association between categorical variables were assessed using chi-square tests, while continuous variable associations were evaluated with two-tailed Student’s t-tests. Statistical significance was determined using p-values (p < 0.05), and 95% confidence intervals (CIs) were reported to assess the precision of the estimates.

**Figure 1.**
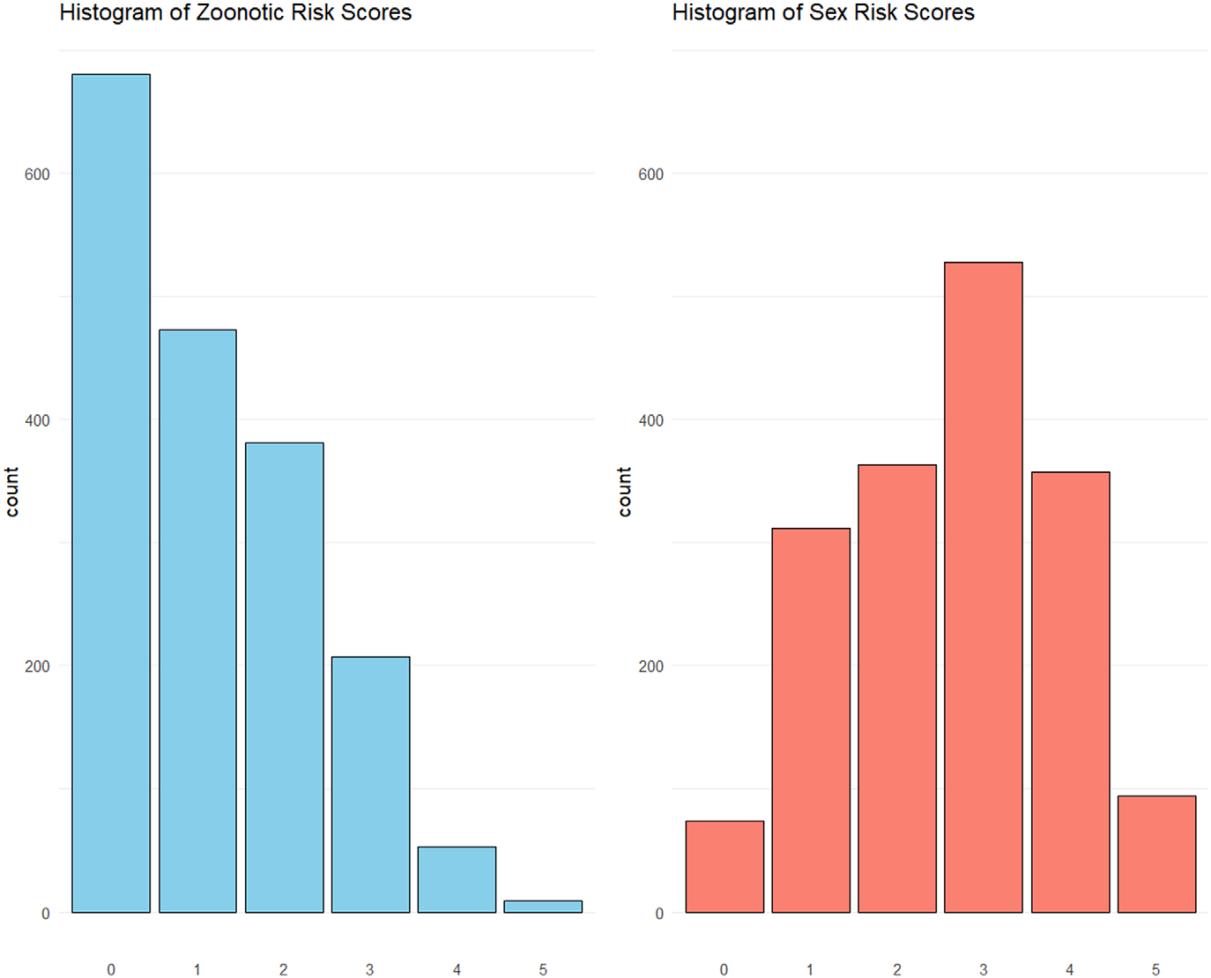
Histograms of Zoonotic (*n* = 1803) and Sex Risk Scores (*n* = 1728). See Supplementary Figures for stratification by age, location, sex and population of interest. Figure 1. long description.

Given the spread of zoonotic and sexual risk factors and unadjusted associations with detected seroreactivity, a 5-point composite score was generated for both zoonotic and sexual risk (Figure 1). Variables included in these scores were selected based on completeness as well as *a priori* understandings of mpox transmission through either zoonotic or human-to-human routes. Zoonotic risk scores were calculated based on the binary responses to the following statements, with each ‘yes’ response equating to one point on the risk scale: (1) Have you ever visited a forested/wooded area?; (2) Have you ever hunted wild animals?; (3) Have you ever prepared a wild animal for cooking?; (4) Have you ever consumed bushmeat?; and (5) Do you work with or interact with wild animals? Similarly, the sexual exposure risk score was calculated with the following responses: (1) Do you have a primary sexual partner? (‘no’ response as one point); (2) In the last three weeks have you had sexual relations with more than one person?; (3) Did you use a condom the last time you had sex? (‘no’ response as one point); (4) Have you ever had sex in exchange for money, drugs, food or accommodation?; and (5) A total partner count greater than the median of the study population (as calculated by total estimated male and female partner counts).

To further understand the relative contributions of predictor variables in explaining seroreactivity results, the R packages ‘tree,’ ‘randomForest’, and ‘party’ [30] were used to generate classification trees and iterative regression models to identify top predictor variables and rankings of predictors contributing to explained seroreactivity (binary outcome, reactive = 1). A final model was employed that consisted of 500 trees, with five covariates randomly selected as splitting candidates for each tree constructed. Model fit was evaluated using an out-of-bag (OOB) error estimate and presentation of the class confusion matrix. Additionally, feature importance was calculated via a variable importance measure (mean decrease in Gini impurity, see Liaw and Wiener for details [30]) and presented alongside a corresponding classification tree. To address class imbalance, an over-weighted model of 350 randomly selected participants including all 53 seroreactivity individuals was run; yet, this only marginally improved the confusion matrix of the model. Finally, logistic regression models to adjust for selected covariates were included to determine potential drivers of seroreactivity with Model 1 including selected demographic characteristics and the collapsed sexual risk score, and Model 2 with the collapsed zoonotic risk score.

## Results

### Overall seroreactivity

A total of 1,823 participants were enrolled between April and June 2024 – 913 (50.1%) from Kinshasa and 910 (49.9%) from Goma. Across specifically recruited sub-populations: 520 MSM (28.5%), 586 SW (32.1%), 110 MSM and SW (6.0%), and 607 ARP individuals (33.3%) were enrolled, with equivalent distributions in the two cities. Serological testing indicated an overall mpox seroprevalence of 2.9% (53/1823) among all participants, with 4.8% reactivity detected among ARP participants. In contrast, reactivity rates were lower among MSM (2.1%) and SW (1.7%) and MSM/SW (2.7%) populations. Seroreactivity was similar across urban centres, with 3.1% detected in Kinshasa and 2.8% in Goma. By antigen, 22 participants were reactive to H3L (1.2%), 17 to B21R.185/186 (0.9%), 12 to B21R.179/180 (0.7%) and 5 to B22R.64/65 (0.3%). (Table 1). Of all 53 reactive individuals, only three respondents were considered reactive to more than one antigen, one reactive to H3L and B21R 180/186, and another two respondents to both B21R antigens (Supplementary Figure S1). No individual was reactive to all four antigens presented.

**Table 1.** Seroreactivity to Selected MPXV-specific Antigens across populations of interest and by location of sampling, *n* = 1823 Table 1. long description.

	Population type, location		Total (%)	Detected seroreactivity									
				H3L		B21R 179/180		B21R 180/186		B22R 64/65		Sum reactivity	
	All participants		1823	22	1.2	12	0.7	17	0.9	5	0.3	53	2.9
	Population of interest												
		Men who have sex with men (MSM)	520 (28.5)	3	0.6	3	0.5	4	0.7	2	0.4	11	2.1
		Sex worker (primary or secondary occupation, SW)	586 (32.1)	4	0.7	3	0.5	3	0.5	1	0.2	10	1.7
		MSM and sex worker	110 (6.0)	1	0.9	0	0.0	2	1.8	0	0.0	3	2.7
		At-risk population (ARP)	607 (33.3)	14	2.3	6	1.0	8	1.3	2	0.3	29	4.8
	Urban center												
		Kinshasa	913 (50.1)	13	1.4	5	0.6	11	1.2	1	0.1	28	3.1
		Goma	910 (49.9)	9	1.0	7	0.8	6	0.7	4	0.4	25	2.8

*Note*: Reported as *n*, % unless otherwise noted.

### Seroreactivity by population demographics and medical history

The median age of participants was 25 years (IR: 21–32), with the majority (46.8%) aged 18–24 years and decreasing representation in older age groups (Table 2). Seroreactivity was significantly associated with age (*p*<0.0001). Participants aged ≥45 years demonstrated markedly higher seroprevalence (11.1–19.2%) compared to younger age groups (2.2–2.9%). When binarizing age by those born before or after the cessation of smallpox vaccination campaigns [31], this difference in seroreactivity is more pronounced with those born pre-1984, reporting 7.8% reactivity (*p* = 0.0001). While the collapsed seroreactivity variable indicates higher reactivity among those born pre-1984 (n = 12/153), by antigen, this is almost exclusively driven by H3L reactivity (*n* = 11, 91.7%) (Supplementary Figure S1).

**Table 2.** MPXV seroreactivity by sample population characteristic. Chi-square *p*-values less than 0.05 are highlighted in green Table 2. long description.

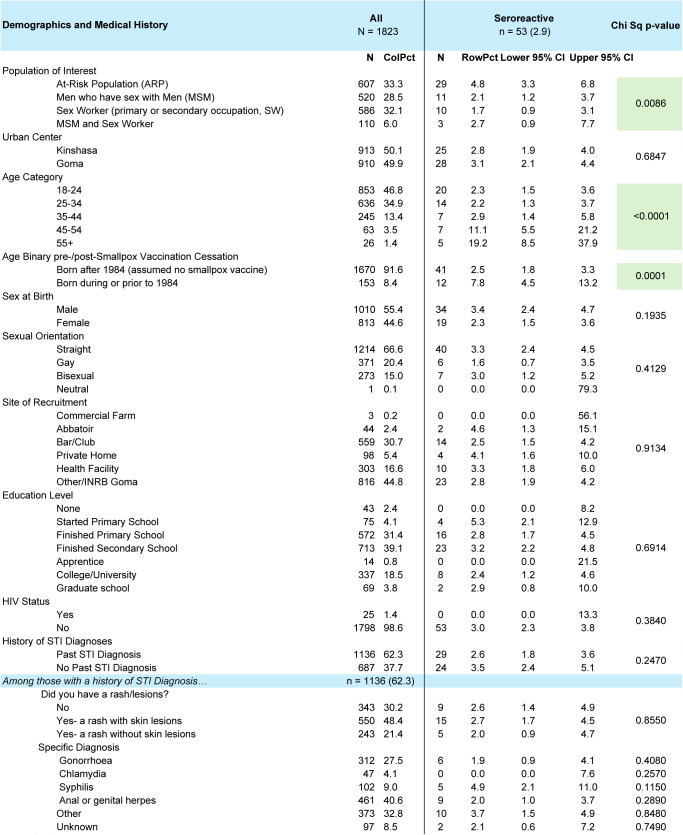

While not statistically significant (*p* = 0.413), heterosexual individuals had a higher overall proportion of seropositivity (3.3%). No significant difference in seroprevalence was observed based on recruitment site (e.g., bars, clubs, health facilities) (*p* = 0.913) or among those reporting zoonotic exposures such as working at abattoirs (*p* = 0.6914). Educational attainment was not significantly associated with seroprevalence (*p* = 0.692), although individuals with only primary education had a slightly higher seroreactivity rate (5.3%) compared to those with secondary or university-level education (2.4–3.2%).

While no significant association was observed (*p* = 0.247) between STI histories and seroreactivity, trends such as higher rates of seroreactivity among those respondents who had no history of past STI diagnosis (3.5% vs. 2.6%, respectively) were observed. Interestingly, among respondents with a self-reported history of STIs (*n* = 1136), those who had reported past syphilis diagnoses were more seroreactive (5/102, 4.9%) than respondents reporting other STIs. Notably, of the 25 individuals who self-identified as HIV-positive, no reactivity was detected.

### Seroreactivity by zoonotic and sexual exposures

Beyond specific demographic characteristics or medical histories, this study sought to evaluate seroreactivity rates by general zoonotic and sexual risk factors (Table 3). Seroreactivity was higher among participants reporting selected wildlife or forest contacts: 22 participants of the 458 who reported ever visiting wooded or forested areas were MPXV seroreactive (4.8%; *p* = 0.0052); 4.3% (22/516; *p* = 0.031) of those who prepared or cooked wild animals, as well as 3.7% of participants who reported bushmeat consumption (37/991; *p* = 0.0232). Occupational animal contact (working at abattoirs or farms, *p* = 0.4595) and reported personal protective equipment use were not significantly associated with seroreactivity.

**Table 3.** MPXV Seroreactivity by selected reported zoonotic and sexual exposures. Chi-square *p*-values less than 0.05 are highlighted in green. Variables indicated by an asterisk (*) are included in the collapsed 5-point zoonotic or sexual risk score Table 3. long description.

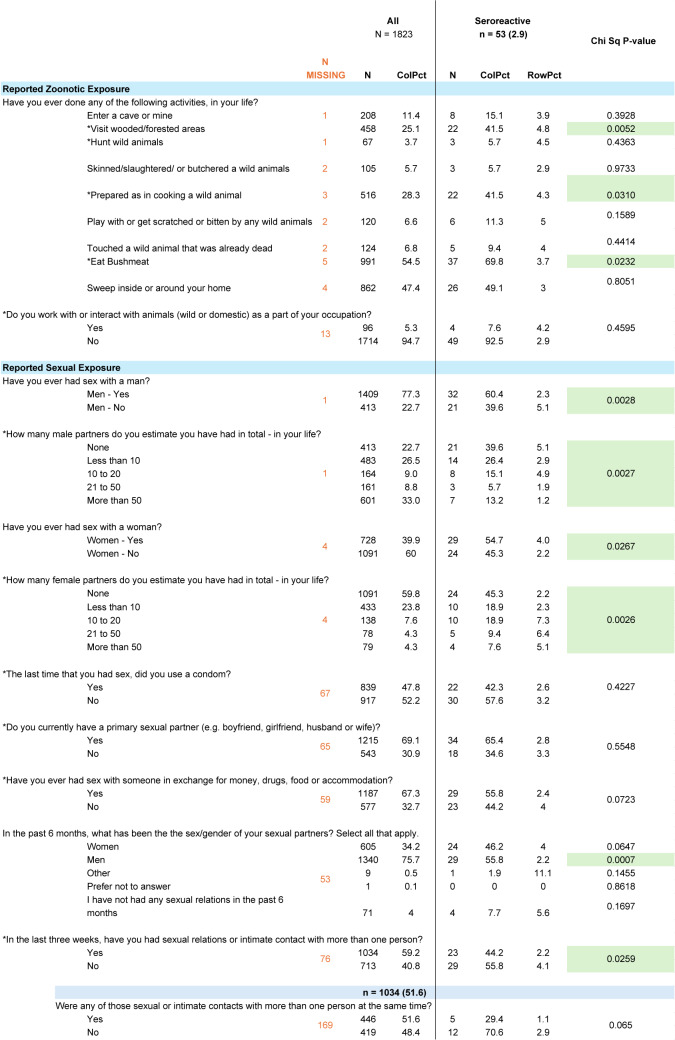

Across all participants, there was a significant association between reporting past sex with both men and women and MPXV seroreactivity: participants who reported ever having had sex with a man had lower rates of reactivity (2.3%), when compared to those who had ever had sex with a woman (4.0%). No significant associations were observed between sexual behaviours such as condom use, group sex, and transactional sex, with seroreactivity.

**Figure 2.**
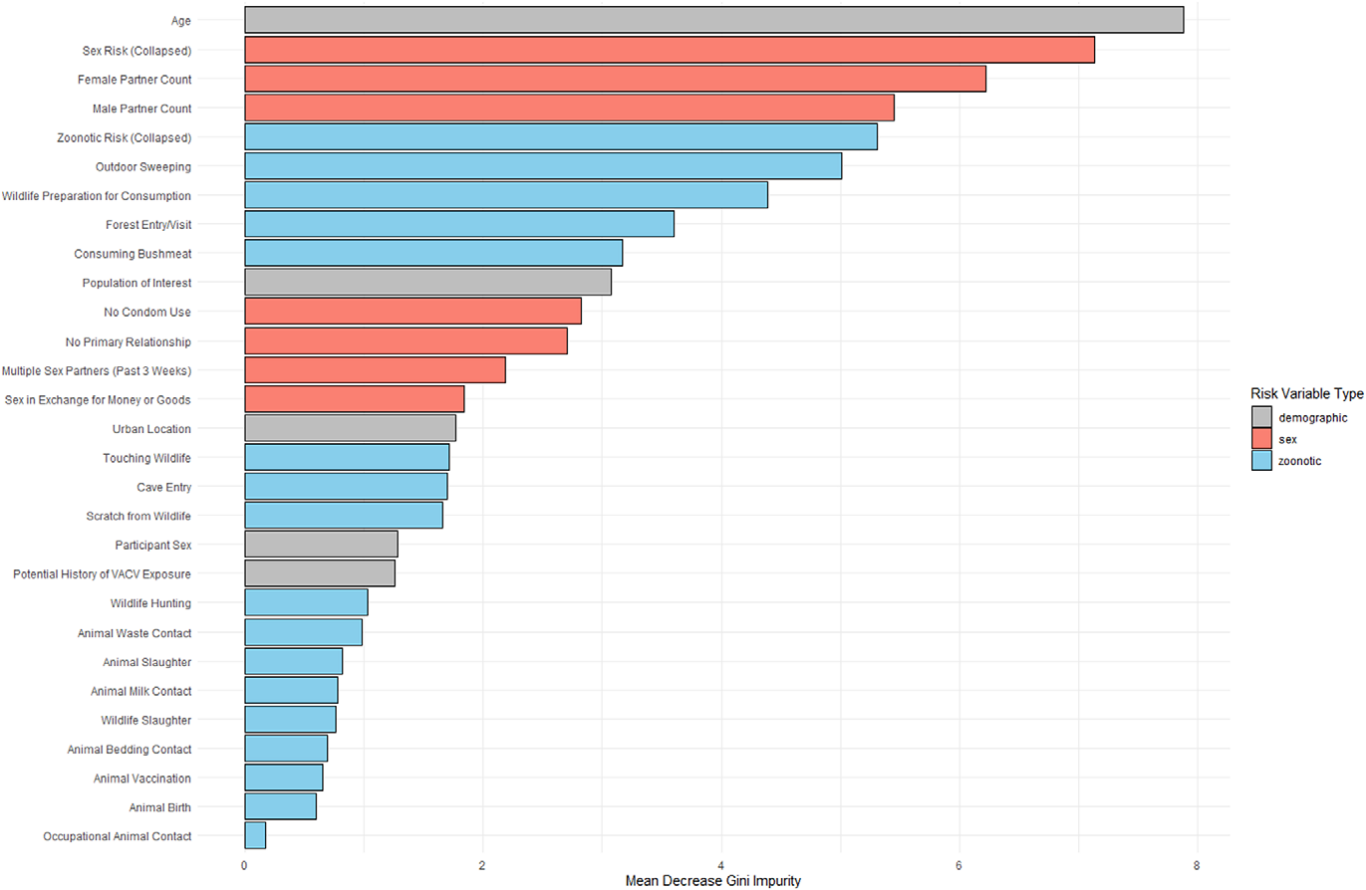
Age as a Key Determinant of Seroreactivity: Feature Importance as generated by Random Forest Model. Feature importance is evaluated as the mean decrease in Gini impurity of each variable in aggregated across all trees of the iterative Random Forest model. Greatest reduction in mean Gini impurity equates to the greatest relative significance of a variable within the context of this model; participant age is ranked as the most influential variable. Figure 2. long description.

**Figure 3.**
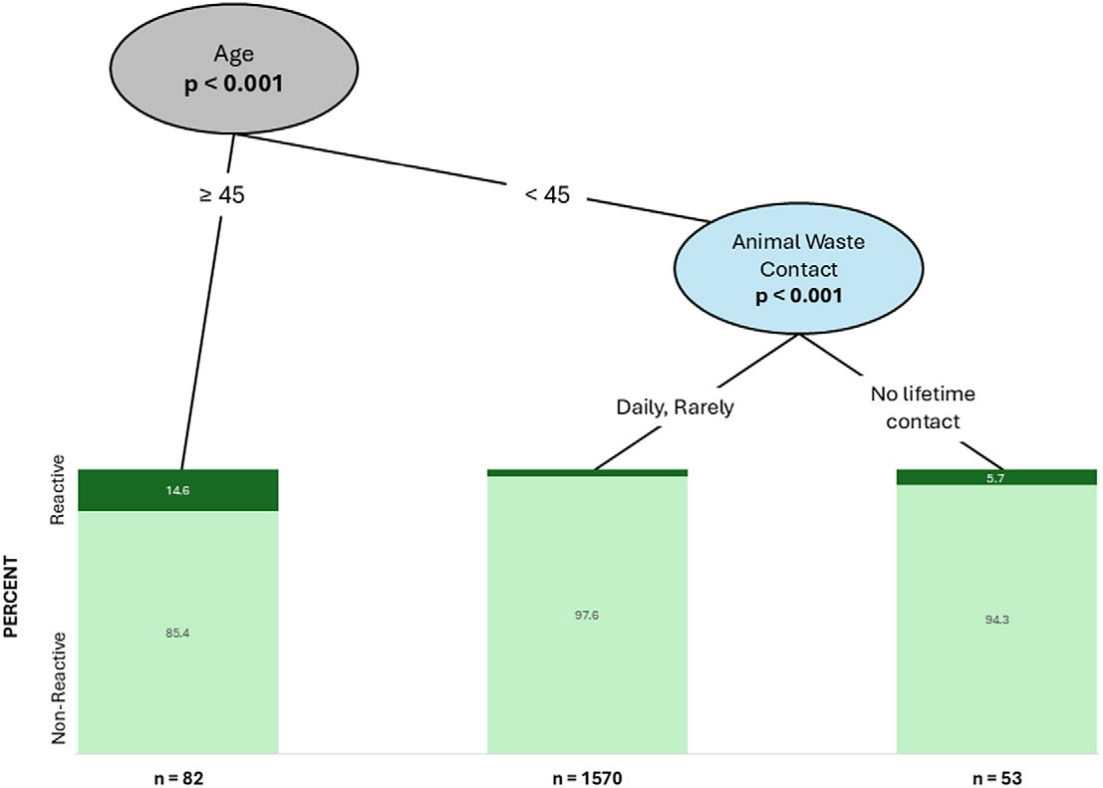
Classification Tree Indicates Age as the Primary Determinant of Seroreactivity. The classification tree plot indicates age as the most informative predictor of seroreactivity with the first significant split (*p* < 0.001) at < 45 and ≥45 years old; among the older participants, 14.6% of this node were seroreactive to MPXV. Figure 3. long description.

The random forest classification model obtained an OOB error rate of 3.1% indicating strong predictive performance (*n* = 1705 complete observations). However, while the class 0 error of this model was 0.0012, the reciprocal class 1 error was 0.981, indicating poor sensitivity of the model to detect true seroreactive cases. Across both the feature importance assessment (Figure 2) and the generated classification tree (Figure 3), the age group split of respondents younger or older than 45 years was identified as the predominant covariate influencing the mpox seroreactive outcome. While handling animal waste is presented as a second node of the classification tree, it is unclear as to why such exposure would have meaningfully contributed to predictive seroreactivity among a younger, urban population who participated in this study, and may simply be acting as a surrogate for more general behavioural/social conditions (i.e., it is possible that this specific question may be indicative of a more general health/hygiene discrepancy between participants or locations).

The random forest approach incorporates potential confounders as splitting variables, yet this statistical method does not provide adjusted estimates for select risk factors and the outcome of binary seroreactivity. To address this, in two separate multivariate logistic regression models adjusting for sex and zoonotic risk, or population type, location, sex, and age, age category was consistently identified as a significant factor associated with seroreactivity (Wald *p*-value = 0.0009, and 0.0021, Table 4). Zoonotic and sexual risk score distributions stratified by key demographic variables are presented in Supplementary Figure S2. In Model 1, when adjusting for the collapsed sexual risk scores, participants aged 45–54 and 55+ had 3.92 and 7.77 times higher odds (CI 1.42–10.81; 2.37–25.52) of seroreactivity than those participants aged 18–24. Additionally, this trend of increased odds in older populations was maintained when adjusting collapsed zoonotic risk scores and other demographic confounders in Model 2: participants aged 45–54 years and 55+ years had 3.49 and 6.11 times higher odds than those participants aged 18–24 (CI: 1.30–9.37; 1.86–20.03). Of note, those individuals with high sexual risk scores did not have reciprocally high zoonotic risk scores (Supplementary Figure S3), indicating true separation between two populations with different possible MPXV exposure routes.

**Table 4.** Adjusted Odds Ratios of Selected Demographic Covariates and Sexual (Model 1) and Zoonotic (Model 2) Risk Scores. Wald p-values less than 0.05 are identified in bold, as are those point estimates considered significant Table 4. long description.

MODEL 1 SEX RISK reactive(x) = age_cat + sex + loc + popcohort + sex_risk	aOR	Lower CI	Upper CI	WALD *p*-value		
	*n* = 1728				Type 3 Wald	*p*-value
**Age**					age_cat2	**0.0009**
18–24	REF				sex	0.2653
25–34	0.866	0.426	1.762	0.6911	location	0.4540
35–44	1.091	0.437	2.726	0.8513	popcohort	0.6418
45–54	**3.923**	**1.423**	**10.818**	**0.0083**	sex_risk	0.4881
55+	**7.774**	**2.368**	**25.521**	**0.0007**		
Sex						
Male	REF					
Female	0.714	0.296	1.725	0.454		
Urban center						
Kinshasa	REF					
Goma	1.158	0.624	2.149	0.6418		
Population cohort						
At risk population	REF					
MSM	0.451	0.192	1.060	0.0677		
MSM and sex worker	0.638	0.163	2.505	0.5195		
Sex worker	0.500	0.158	1.587	0.2396		
5-Point sex risk (Multiple partners in three weeks, QPQ Sex, Total partner count > median, no condom use, non-monogamous/no primary partner)						
0	REF					
1	0.326	0.105	1.010	0.0520		
2	0.677	0.241	1.898	0.4580		
3	0.542	0.175	1.672	0.2863		
4	0.623	0.177	2.196	0.4620		
5	0.593	0.102	3.464	0.5619		

MODEL 2 ZOO RISK model reactive(x) = age_cat + sex + loc + popcohort + zoo_risk	*n* = 1803				Type 3 Wald	*p*-value
Age					age_cat2	**0.0021**
18–24	REF				sex	0.3341
25–34	0.773	0.381	1.568	0.4761	location	0.8961
35–44	0.964	0.389	2.390	0.9375	popcohort	0.2465
45–54	**3.493**	**1.303**	**9.366**	**0.0129**	zoo_risk	**0.0204**
55+	**6.109**	**1.863**	**20.025**	**0.0028**		
Sex						
Male	REF					
Female	0.659	0.282	1.537	0.3341		
Urban center						
Kinshasa	REF					
Goma	0.96	0.518	1.778	0.8961		
Population cohort						
At risk population	REF					
MSM	0.491	0.223	1.085	0.0788		
MSM and sex worker	0.63	0.169	2.356	0.4925		
Sex worker	0.585	0.225	1.519	0.2709		
5-point zoonotic risk (working with animals, entering forest, preparing wild meat, eating bushmeat and hunting wild animals)						
0	REF					
1	1.691	0.697	4.101	0.2454		
2	**3.973**	**1.761**	**8.963**	**0.0009**		
3	**3.316**	**1.261**	**8.724**	**0.0151**		
4	2.357	0.467	11.91	0.2994		
5	<0.001	<0.001	>999.999	0.988		

*Note:* Wald *p*-values less than 0.05 are identified in bold, as are those point estimates considered significant.

## Discussion

This serosurvey of high-risk urban populations provides a unique cross-sectional estimate of mpox exposure rates prior to the sustained human-to-human transmission of the virus in Kinshasa and Goma first reported in the later part of 2024. While overall seroprevalence was low (2.9%), patterns across demographic and behavioural groups offer important insights into evolving transmission dynamics.

The sample collection period of this study suggests that the detected infections likely resulted from sporadic zoonotic introductions and small, self-limiting clusters – similar to those historically documented in Central Africa – rather than the prolonged human-to-human transmission chains now observed in urban centres of DRC [25]. These initial transmission dynamics may be best described as stuttering chains with an initial spillover event seeding weak human-to-human spread [32–34]. Specifically, in Kinshasa, the first case of Clade Ib MPXV was detected in July 2024 and large-scale detection of sustained human transmission was not confirmed until mid-August 2024, well after the sampling period of this study [25, 35]. Similarly, the first cases of Clade Ib MPXV were not confirmed in Goma until May-June 2024, while this study was being conducted [24]. Given the general timeline of antibody development, it is highly likely that all detected seroreactivity predates the confirmed introduction of Clade Ib MPXV in both cities. While mpox in endemic regions has been traditionally attributed to rural wildlife-to-human spillover, our findings show that such introductions may also occur in urban contexts, potentially seeding emerging transmission networks.

Seroprevalence varied across demographic groups, with older age strongly associated with seroreactivity. However, the higher prevalence among older participants could reflect cumulative lifetime exposure or residual immunity from historic smallpox vaccination campaigns. Moreover, when dividing the cohort by those born before and after 1984 (presumed vaccination), all but one of the 12 individuals identified as reactive were specifically reactive to the H3L antigen. While smallpox was officially declared as eradicated in 1980, there has been evidence of continued sporadic vaccination campaigns in the DRC up until 1984 [31]. Here, we specifically chose birth year of 1984 as a more conservative cut off for potential exposure to the vaccina-based vaccine. Notably, the age split at 45 years as indicated by the regression tree aligns with a 1980 birth year cut point, potentially indicating that exposure among this group (the same 12 reactive individuals as identified by the 1984 cut point) is instead driven by historic orthopoxvirus vaccination.

The H3L envelope protein is a well conserved region across the *Poxviridae* family, including vaccinia virus (VACV) [29]. As this antigenic target was included as a component of the binary reactivity status, a history of VACV exposure – whether via vaccination or natural infection – may instead be driving this observed association. This potential cross-reactivity with vaccinia-derived immunity should not be understated as the potential true mechanism driving higher reactivity rates among older participants. However, as we did not specifically collect vaccinia vaccination history, respondent age serves as a proxy for this exposure, where those over 45 years old are highly likely to have been vaccinated as part of smallpox eradication practices.

Yet, among a separate cohort of individuals with differential orthopoxvirus exposures, only 6/26 smallpox vaccines were reactive to H3L (unpublished data). Previous studies have indicated that waning smallpox immunity in DRC has gradually eroded cross-protective immunity, thereby increasing the proportion of immunologically naïve individuals susceptible to mpox [5]. As the younger MSM and SW of this cohort are unlikely to have been vaccinated for smallpox, these populations are likely to suffer additional disease burden as human-to-human mpox transmission persists. The lower seroreactivity rates, and by extension, immune-naive status among MSM and SW identify MSM and SW populations as high priority groups for mpox vaccination campaigns.

The highest seroreactivity was detected among the sampled ARP (farm workers, non-MSM, and non-SW) (4.8%), a pattern consistent with traditional pathways of mpox exposure via animal-to-human contact. Although age was identified as the most predominant predictor of seroreactivity – both by the random forest and generalized linear regression model approaches – notable associations were also observed with self-reported behaviours indicative of zoonotic risks: specifically, bushmeat consumption and visiting forested areas. Interestingly, these activities are typically considered to be elements of rural exposure profiles; yet over 50% of respondents in Kinshasa and Goma had reported consuming wild animals at least once in their lifetime. This finding suggests that the boundaries between rural and urban exposure routes may be increasingly blurred, emphasizing the interconnected nature of zoonotic risk in the urban landscape of the DRC. Future investigations should build off these findings to better define which key factors may be driving this zoonotic risk among this ARP, as well as sampling of a true urban general population to establish a more universal urban seroreactivity threshold irrespective of sub-population by occupational or behavioural classifications. Furthermore, the sampling design of this approach further limits generalizability of these seroreactivity rates to the larger urban population; these results are limited in their applicability beyond those specific cohorts recruited for this study.

Unlike similar studies conducted in Kenya, which identified strong seropositivity among SW and MSM communities in Nairobi [23], sexual risk behaviours and identification as SW or MSM were not identified as drivers of mpox seroreactivity in Kinshasa and Goma. However, these differences may be attributed to the sampling strategy employed – prospective as compared to retrospective from convenience sampling – antibody targets of the serologic assay, and other additional variables including community-specific behavioural differences. Statistical associations were only observed between reactivity and reported lifetime partner counts and frequent intimate contacts, yet these exposures did not supersede age as drivers of the reactivity outcome when assessed by the classification model. Sexual behaviour patterns suggest that mixed sexual networks may facilitate early mpox spread. Heterosexual men and MSM reporting vaginal intercourse showed higher seroprevalence, indicating potential bridging between heterosexual and MSM networks. This differs from the 2022 Clade IIb global outbreak, which was largely confined to MSM populations [9].

While our iterative regression model achieved a low average error estimate, ultimately this approach had poor sensitivity in detecting seroreactive cases, most likely due to class imbalance. Such machine-learning approaches (e.g., partitioned tree classifications, random forests) can always be improved with the inclusion of additional variables and response data; ongoing work is currently being conducted to re-enrol these same target populations to capture additional predictor variables and reassess seroreactivity.

As with any cross-sectional study design, this approach limits the ability to differentiate between recent and historic mpox exposure; lifetime exposure questions may also be subject to recall and reporting biases, leading to a possible over- or under-representation of the true exposure histories of participants. Of note, when stratifying zoonotic and sexual risk scores, older participants reported lower scores for both metrics. This could be yet another example of recall bias, and further highlights how the age-stratified populations differ significantly in outcome and reported exposures. Future longitudinal sampling of these vulnerable urban populations, especially following the sustained human-to-human transmission reported in late 2024, would help to further elucidate which specific behavioural, sexual and zoonotic risks may be contributing to mpox spread in urban areas the DRC. Another limitation of this study is the binary approach to seroreactivity, while the B21R and B22R antigenic targets are MPXV-specific, the inclusion of H3L reactivity as a component of the MPXV reactivity definition may overestimate exposure. While supplementary data has demonstrated most reactivity (50/53 respondents) was to one antigen of the panel. Of those reactive individuals, 22 were uniquely reactive to the highly conserved H3L antigen. As age was found to be highly associated with detected seroreactivity, this may be indicative of a decrease in the immune repertoire with age, where only those H3L-specific antibodies are conserved as participants age [36]. Furthermore, a history of mild or subclinical infections (likely undetected by the health reporting systems) may also explain the detection of single-antigen reactivity. Finally, given the reactivity cut-off approach employed, there is a possibility that some reactivity to secondary targets may exist, yet below the threshold as set previously. Further antigen-specific analysis should be conducted to better elucidate potential MPXV-specific exposure.

Our results suggest low-level exposure to mpox among a subset of vulnerable urban populations. Of note, the four-plex MPXV serologic assay employed in this study has reported good sensitivity and high specificity to detect antibodies both early in infection, but also showed antibody reactivity decline overtime, up to two years after infection [18, 37]. This facet of the assay functionality is in line with our conclusion that those detected seroreactive individuals may have higher reactivity rates with age – whether that be historic orthopoxvirus vaccination status, or more simply increased exposure frequency over time as an inhabitant of an mpox-endemic region. These results define a potential exposure rate to mpox, specifically among populations suspected to be at higher risk in an urban setting.

As Clade I MPXV continues to rapidly evolve, with increasing evidence of sustained human-to-human transmission of both Clades Ia and Ib [25], these results can inform context-specific behavioural and demographic vulnerabilities to guide early detection and public health intervention. While some transmission characteristics may be environmentally, behaviourally, or culturally bespoke, the MPXV transmission cycle itself is not unique to DRC, and by identifying particularly vulnerable populations in one setting, results are likely to help identify similar ARPs in others. Baseline serosurveys such as this are crucial for understanding the foundations of emerging epidemics. By characterizing pre-outbreak exposures and the complex interplay of zoonotic and behavioural risk factors among key populations in an endemic setting, this work provides a vital reference point for interpreting current and future urban mpox outbreaks – both in DRC, and abroad.

## Supporting information

10.1017/S0950268826101861.sm001 Mukadi et al. supplementary material Mukadi et al. supplementary material

## Data Availability

Data will be made available upon reasonable request to the corresponding author.

## Supplementary material

The supplementary material for this article can be found at http://doi.org/10.1017/S0950268826101861.

## Long descriptions

### Figure 1 Long description

A two-panel figure displaying frequency distributions.

Left Panel: Histogram of Zoonotic Risk Scores.

- The Y-axis is labeled count, ranging from 0 to 600 with increments of 200.

- The X-axis represents scores from 0 to 5.

- The data is shown in light blue bars.

- Score 0 has the highest count, exceeding 600.

- The counts decrease steadily as the score increases, showing a right-skewed distribution.

- Score 1 is approximately 480, score 2 is approximately 380, score 3 is approximately 210, score 4 is approximately 60, and score 5 is near 10.

Right Panel: Histogram of Sex Risk Scores.

- The Y-axis is labeled count, ranging from 0 to 600 with increments of 200.

- The X-axis represents scores from 0 to 5.

- The data is shown in coral-colored bars.

- The distribution is roughly bell-shaped, peaking at score 3 with a count of approximately 520.

- Score 0 is approximately 80, score 1 is approximately 310, score 2 is approximately 360, score 4 is approximately 350, and score 5 is approximately 100.

Navigate back to Figure 1.

### Table 1 Long description

The table presents detected seroreactivity for 1823 total participants. The columns are categorized by Population type and location, Total count and percentage, and specific seroreactivity markers: H 3 L, B 21 R 179/180, B 21 R 180/186, B 22 R 64/65, and Sum reactivity. Each marker includes a count and a percentage.

* All participants: Total 1823. H 3 L: 22 (1.2%). B 21 R 179/180: 12 (0.7%). B 21 R 180/186: 17 (0.9%). B 22 R 64/65: 5 (0.3%). Sum reactivity: 53 (2.9%).

Population of interest breakdown:

* Men who have sex with men (M S M): Total 520 (28.5%). Sum reactivity: 11 (2.1%).

* Sex worker (S W): Total 586 (32.1%). Sum reactivity: 10 (1.7%).

* M S M and sex worker: Total 110 (6.0%). Sum reactivity: 3 (2.7%).

* At-risk population (A R P): Total 607 (33.3%). Sum reactivity: 29 (4.8%).

Urban center breakdown:

* Kinshasa: Total 913 (50.1%). Sum reactivity: 28 (3.1%).

* Goma: Total 910 (49.9%). Sum reactivity: 25 (2.8%).

Navigate back to Table 1.

### Table 2 Long description

The table is organized into five columns: Characteristic, Total N, Seropositive n, Seropositive percentage, and p-value. Total for all groups is 1823.

* Population of Interest: . At-Risk Population (n=607, 33.3%), Men who have sex with Men (n=520, 28.5%), Sex Worker (n=586, 32.1%), MSM and Sex Worker (n = 110, 6.0%). p-value is less than 0.001 (highlighted green).

* Sex: . Male (n=1010, 55.4%), Female (n=813, 44.6%). p-value is 0.19.

* Age Group: . 18-24 (n=853, 46.8%), 25-34 (n=636, 34.9%), 35 to 44 (n=245, 13.4%), 45-54 (n=63, 3.5%), 55 plus (n = 26, 1.4%). p-value is <0.001 (highlighted green).

* Smallpox Vaccination Status: Born after 1984 (assumed no smallpox vaccination) (n=1670, 91.6%), Born during or prior to 1984 (n=153, 8.4%). p-value is 0.0001 (highlighted green).

* History of Rash Illness: Total 1136. Yes, with lessions (n=550, 48.4%), Yes, no lesions (n = 243, 21.4%) No (n=343, 30.2%). p-value is 0.855.

Navigate back to Table 2.

### Table 3 Long description

The table is organized into four columns: Variable, Total Population (N = 1823), Seroreactive (n = 53), and P-value.

Under the Zoonotic Exposure category:

- Butchered or handled bushmeat: Total 105 (5.7 percent), Seroreactive 3 (2.9 percent), P-value 0.97.

- Scratched or bitten by by any wild animals: Total 120 (6.6 percent), Seroreactive 6 (5.0 percent), P-value 0.44.

Under the Sexual Exposure category:

- Multiple sexual partners in the last three weeks: Total 1034 (59.2 percent), Seroreactive 23 (3.3 percent), P-value 0.0259.

Navigate back to Table 3.

### Figure 2 Long description

The x-axis represents Mean Decrease Gini Impurity ranging from 0 to 8. The y-axis lists 28 risk variables. A legend on the right identifies three Risk Variable Types: demographic (gray), sex (red), and zoonotic (blue).

From top to bottom, the variables and their approximate values are:

* Age (demographic): 7.9

* Sex Risk (Collapsed) (sex): 7.1

* Female Partner Count (sex): 6.2

* Male Partner Count (sex): 5.4

* Zoonotic Risk (Collapsed) (zoonotic): 5.3

* Outdoor Sweeping (zoonotic): 5.0

* Wildlife Preparation for Consumption (zoonotic): 4.4

* Forest Entry/Visit (zoonotic): 3.9

* Consuming Bushmeat (zoonotic): 3.2

* Population of Interest (demographic): 3.1

* No Condom Use (sex): 2.8

* No Primary Relationship (sex): 2.7

* Multiple Sex Partners (Past 3 Weeks) (sex): 2.2

* Sex in Exchange for Money or Goods (sex): 1.8

* Urban Location (demographic): 1.7

* Touching Wildlife (zoonotic): 1.7

* Cave Entry (zoonotic): 1.6

* Scratch from Wildlife (zoonotic): 1.6

* Participant Sex (demographic): 1.3

* Potential History of V A C V Exposure (demographic): 1.3

* Wildlife Hunting (zoonotic): 1.0

* Animal Waste Contact (zoonotic): 0.7

* Animal Slaughter (zoonotic): 0.6

* Animal Milk Contact (zoonotic): 0.6

* Wildlife Slaughter (zoonotic): 0.5

* Animal Bedding Contact (zoonotic): 0.4

* Animal Vaccination (zoonotic): 0.4

* Animal Birth (zoonotic): 0.3

* Occupational Animal Contact (zoonotic): 0.1

Navigate back to Figure 2.

### Figure 3 Long description

The decision tree begins at a top grey oval node labeled Age with a p-value less than 0.001.

Two branches descend from this node.

1. The left branch is for age greater than or equal to 45. It leads directly to a terminal stacked bar chart with a sample size n equals 82. This chart shows 14.6 percent Reactive (dark green) and 85.4 percent Non-Reactive (light green).

2. The right branch is for age less than 45. It leads to a second blue oval node labeled Animal Waste Contact with a p-value less than 0.001.

From the Animal Waste Contact node, two further branches descend.

1. The left branch is for Daily or Rarely contact. It leads to a terminal stacked bar chart with a sample size n equals 1570. This chart shows 2.4 percent Reactive (represented by a thin dark green sliver) and 97.6 percent Non-Reactive (light green).

2. The right branch is for No lifetime contact. It leads to a terminal stacked bar chart with a sample size n equals 53. This chart shows 5.7 percent Reactive (dark green) and 94.3 percent Non-Reactive (light green).

The vertical Y-axis for all bar charts is labeled PERCENT, with categories Reactive and Non-Reactive indicated on the far left.

Navigate back to Figure 3.

### Table 4 Long description

The table presents two multivariable models.

Model 1 (Sexual Risk) with n = 1728:

- Age: Significant increase in risk for ages 45 to 54 (a O R 3.923) and 55 plus (a O R 7.774) compared to the 18 to 24 reference group.

- Sex: Females (a O R 0.714) compared to Male reference.

- Urban Center: Goma (a O R 1.158) compared to Kinshasa reference.

- Population Cohort: M S M (a O R 0.451), M S M and sex worker (a O R 0.638), and sex worker (a O R 0.500) compared to at-risk population reference.

- 5-Point Sex Risk: Scores 1 through 5 show a O R values ranging from 0.326 to 0.677 compared to a score of 0 reference.

Model 2 (Zoonotic Risk) with n = 1803:

- Age: Significant increase for ages 45 to 54 (a O R 3.493) and 55 plus (a O R 6.109).

- Sex: Females (a O R 0.659).

- Urban Center: Goma (a O R 0.96).

- Population Cohort: M S M (a O R 0.491), M S M and sex worker (a O R 0.63), and sex worker (a O R 0.585).

- 5-Point Zoonotic Risk: Significant increase for score 2 (a O R 3.973, p = 0.0009) and score 3 (a O R 3.316, p = 0.0151) compared to score 0 reference.

Type 3 Wald p-values indicate age_cat2 is significant in both models (p = 0.0009 and p = 0.0021 respectively), and zoo_risk is significant in Model 2 (p = 0.0204).

Navigate back to Table 4.
