# Supplementary material for: Mpox seroprevalence among sex workers and men who have sex with men preceding sustained urban outbreak in the Democratic Republic of the Congo
Source: Epidemiol Infect. 2026 Jun 23;154:e107. doi: 10.1017/S0950268826101861 (PMC13459010; doi:10.1017/S0950268826101861)
Supplement: Supplementary file 1 [file S0950268826101861sup001.docx]

**Supplementary Table 1A. Co-Reactivity to all four antigens of the multiplex immunoassay among all cohort members (n = 1823).**

**Supplemental Table 1B. With age as a proxy for smallpox vaccine exposure (VACV), Co-reactivity of all four antigens among cohort members born before 1984 (n = 153)**

**Supplementary Table 1C. Co-Reactivity to all four antigens of the multiplex immunoassay among cohort members born after 1984 (n = 1670).**

**Supplemental Figure 1. Multi-Target Seroreactivity among all cohort members (n = 53), those born pre=1984 (n = 12) and those born post-1984 (n = 41)**

**Supplemental Figure 2A. Zoonotic and Sexual Risk scores stratified by urban center.**

**Supplemental Figure 2B. Zoonotic and Sexual Risk scores stratified by population of interest.**

**Supplemental Figure 2C. Zoonotic and Sexual Risk scores stratified by respondent sex at birth.**

**Supplemental Figure 2D. Zoonotic and Sexual Risk scores stratified by age category.**

**Supplementary Figure 3. Overlap of Sexual and Zoonotic Risk Scores.** Individual counts are presented in each cell.
